# Supplementary material for: Melatonin-Mediated Regulation of Growth and Antioxidant Capacity in Salt-Tolerant Naked Oat under Salt Stress
Source: Int J Mol Sci. 2019 Mar 7;20(5):1176. doi: 10.3390/ijms20051176 (PMC6429221; doi:10.3390/ijms20051176)
Supplement: Supplementary file 1 [file ijms-20-01176-s001.pdf]

# Supplementary Materials

## Melatonin-mediated regulation of growth and antioxidant capacity in salt-tolerant naked oat under salt stress

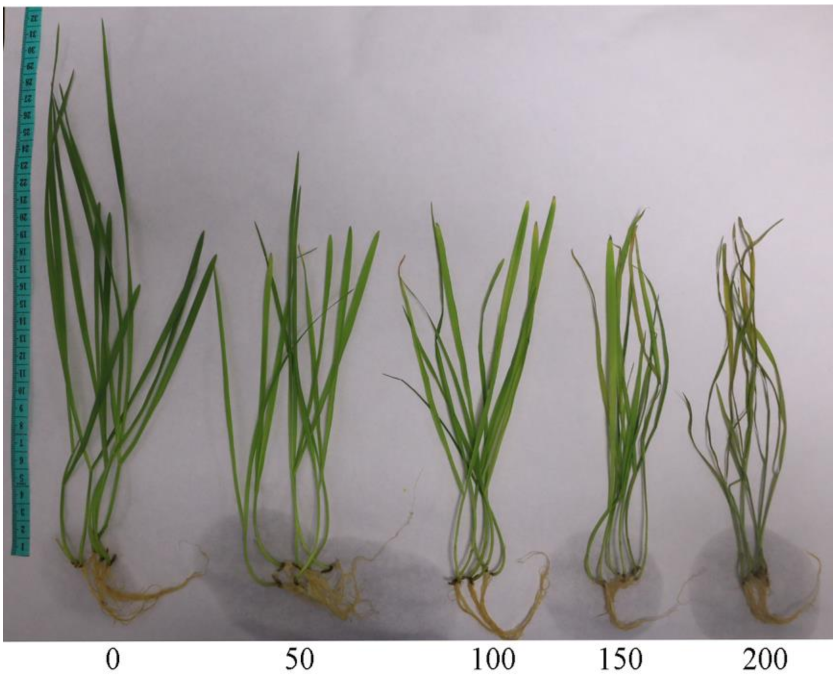


**Figure 1.** Effect of different concentrations of salt stress treatment on the growth of naked oat seedlings. Photograph was taken after salt stress for seven days. Control seedlings without salt pretreatment, 0; seedlings pretreated with NaCl at concentrations of 50 mM (50), 100 mM (100), 150 mM (150), and 200 mM (200).
